# ProDFace: A web-tool for the dissection of protein-DNA interfaces

**DOI:** 10.3389/fmolb.2022.978310

**Published:** 2022-09-06

**Authors:** Arumay Pal, Pinak Chakrabarti, Sucharita Dey

**Affiliations:** ^1^ School of Bioengineering, Vellore Institute of Technology, Bhopal, India; ^2^ Department of Biochemistry, Bose Institute, Kolkata, India; ^3^ Department of Bioscience and Bioengineering, Indian Institute of Technology Jodhpur, Karwar, India

**Keywords:** protein-DNA interactions, sequence conservation, interface core and rim, hydrogen bond, docking, Protein-DNA complexes

## Abstract

Protein-DNA interactions play a crucial role in gene expression and regulation. Identifying the DNA binding surface of proteins has long been a challenge–in comparison to protein-protein interactions, limited progress has been made in the development of efficient DNA binding site prediction and protein**-**DNA docking methods. Here we present ProDFace, a web tool that characterizes the binding region of a protein-DNA complex based on amino acid propensity, hydrogen bond (HB) donor capacity (number of solvent accessible HB donor groups), sequence conservation at the interface core and rim region, and geometry. The program takes as input the structure of a protein-DNA complex in PDB (Protein Data Bank) format, and outputs various physicochemical and geometric parameters of the interface, as well as conservation of the interface residues in the protein component. Values are provided for the whole interface, and after dissecting it into core and rim regions. Details of water mediated HBs between protein and DNA, potential HB donor groups present at the binding surface of protein, and conserved interface residues are also provided as downloadable text files. These parameters can be useful in evaluating and validating protein-DNA docking solutions, structures derived from simulation as well as solutions from the available prediction tools, and facilitate the development of more efficient prediction methods. The web-tool is freely available at structbioinfo.iitj.ac.in/resources/bioinfo/pd_interface *.*

## Introduction

Protein-nucleic acid recognition plays an essential role in all mechanisms of gene expression and control. Over the last two decades several groups have attempted to study and characterize the DNA-binding region that is crucial for recognition (Steitz 1990; Jones et al., 1999; Nadassy et al., 1999; Ahmad et al., 2004; Biswas et al., 2009; Corona and Guo 2016). Several features studied, such as the amino acid composition (Jones et al., 1999; Nadassy et al., 1999; Ahmad et al., 2004; Biswas et al., 2009), the conservation of amino acid residues as well as base-pairs (Luscombe and Thornton 2002; Mirny and Gelfand 2002; Kuznetsov et al., 2006; Ahmad et al., 2008), interactions at specific amino acid-base level (Mandel-Gutfreund and Margalit 1998), hydrophobic patches, non covalent interactions at atomic level (Luscombe et al., 2001), electrostatic potential (Jones et al., 2003; Stawiski et al., 2003), ionization state of amino acid side chains (the side chain pKa value) (Wang and Brown 2006), asymmetric distribution of electrostatic charge (Pal and Levy 2020) suggest that the amino acids at the interface possess characteristics that distinguish them from the rest of the protein. Thermodynamic and structural data on protein-DNA interactions have been combined to explore relationships between free energy, sequence conservation and structural cooperativity (Ahmad et al., 2008). Polar interactions at the interface also have an important role in binding. The importance of water molecules in protein-DNA interactions has been recognized, though to what extent they contribute to the binding specificity is still not clear (Reddy et al., 2001; Sarai and Kono 2005).

While there are a number of web servers dealing with the structural features of protein-protein interactions, such programs are almost non-existent for protein-DNA interactions, those few available earlier like WebPDA (Kim and Guo 2009), are now obsolete. Even databases like BIPA, PDIdb (Lee and Blundell 2009; Norambuena and Melo 2010) are either completely not functional now or outdated. DNAProDB (Sagendorf et al., 2017) is a more recently developed database in this area that provides precomputed structural features of protein-DNA complexes taken from PDB (until 2019). However, the interactive DNAProDB is mostly dedicated to give contact maps of interacting residues, given a protein-DNA complex. Similar to DNAProDB, COCOMAPS (Vangone et al., 2011) is another web-tool that also gives contact maps of interacting residues, given a protein-protein/DNA/RNA complex. A database named ProNAB (Harini et al., 2022) has been recently developed that provides experimentally validated thermodynamic parameters like dissociation constant (Kd), binding free energy (ΔG) and change in binding free energy upon mutation (ΔΔG) values, secondary structure and accessible surface area (ASA), for ∼20,000 protein-DNA/RNA complexes.

In our previous work (Dey et al., 2012), we have developed a set of parameters, based on a thoroughly curated non-redundant dataset of 130 protein-DNA interfaces, that could identify DNA binding region, both individually and in combination, to a high degree of accuracy (90.5% for the bound structures and 93.6% for the unbound form of the proteins). In this work, we have developed a web tool, ProDFace by tuning those parameters, viz., the number of evolutionary conserved residues (*N*_*cons*_), the number of potential hydrogen bond donors (*D*_*p*_) and residue propensity score (*R*_*p*_), for community use. We have also integrated other important physicochemical and geometric features from two of our earlier developed web-tools ProFace (Saha et al., 2006), now hosted at *structbioinfo.iitj.ac.in/resources/bioinfo/interface* and PRICE (Guharoy et al., 2011), now hosted at structbioinfo.iitj.ac.in/resources/bioinfo/PPI_energetics, that deal with protein-protein interactions. With the availability of increasing amount of experimentally derived binding data such as dissociation constant (Kd) and binding free energy (ΔG) (Harini et al., 2022), our protein-DNA interface parameters can be used to correlate statistically derived features with experimental data.

Hence, we anticipate that ProDFace would be useful for analysing the increasing number of DNA-binding proteins and that the features it provides can be a useful implementation in the development of protein-DNA docking algorithms.

## Implementation of the program

**FIGURE 1:**
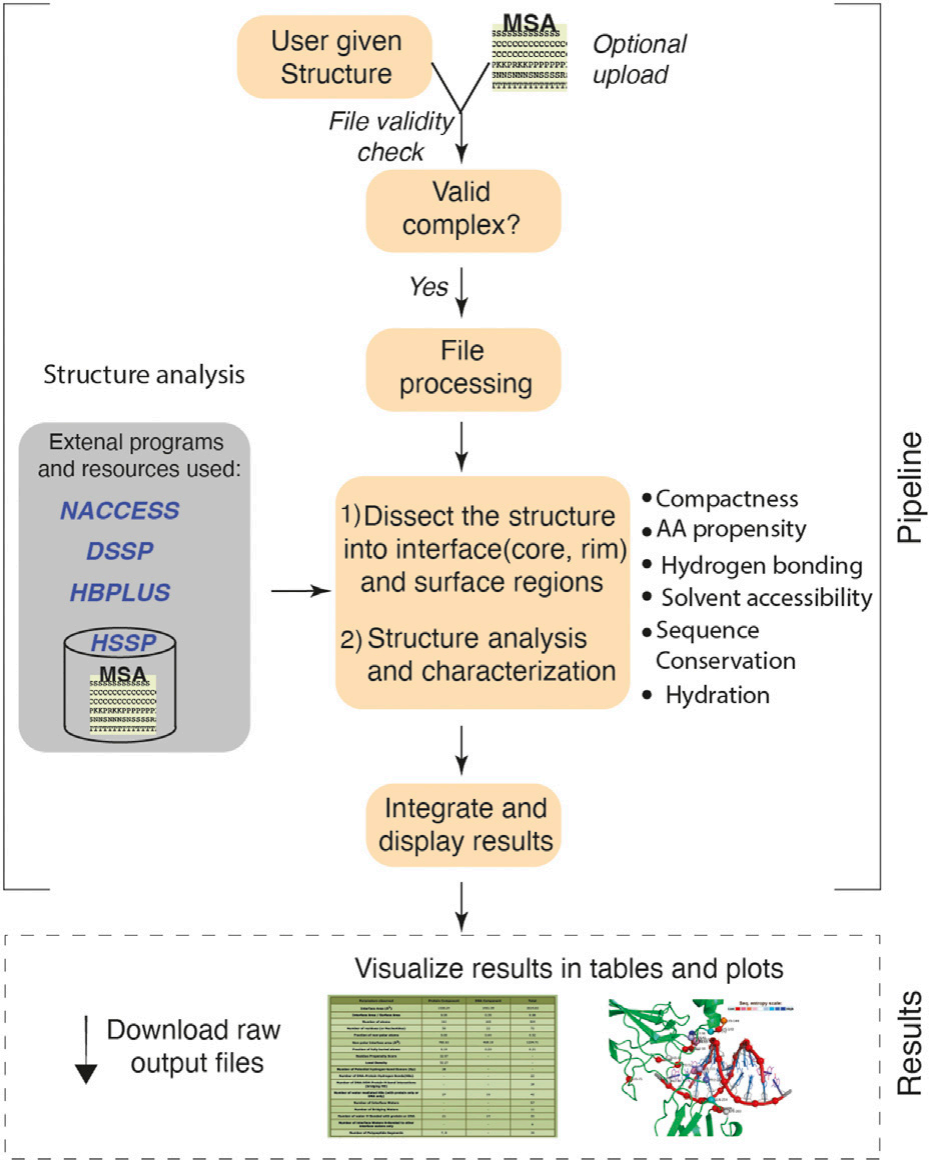
Workflow of ProDFace. The user uploads a query structure in PDB format. The protein-DNA binding region i.e. the interface and the rest of the surface region are identified for the whole complex, as well as for the protein and the DNA components. Structural, geometrical and physico-chemical properties of the interface region are calculated. Also, the interface hydration is analyzed. The interface is dissected into core and rim regions depending on their solvent accessibility (Guharoy and Chakrabarti, 2005) and the sequence conservation within these areas is calculated. All these properties have been described previously (Dey et al., 2012). The conserved residues are displayed in a separate plot.

Given the atomic coordinates of a protein-DNA complex, ProDFace does data mining using a collection of in house programs running at the backend, and extracts various features that helps one to study the nature of the binding region. In addition, some free softwares used are: NACCESS (Hubbard, 1992) for the calculation of accessible surface area, HBPLUS (McDonald and Thornton 1994) for locating hydrogen bonds, DSSP (Kabsch and Sander 1983) for defining the secondary structural elements of proteins, and SURFNET (Laskowski 1995) for transforming the coordinates of the interface atoms along their principal axes and then projecting down the shortest axis. The work flow of the ProDFace pipeline is shown pictorially in Figure 1.

### ProDFace input

The user provides two main pieces of information to the program. First, a protein-DNA complex structure file in PDB (Berman et al., 2000) format is uploaded. The structure may contain one or more protein as well as DNA chains. The second information needed is the chain identifiers both for the protein and DNA, to be used for calculating the interface.​​ The user can also provide a self generated multiple sequence alignment (MSA) of the query protein with its homologs, which is optional. If no alignment file is provided, the program generates the alignment using HSSP database (homology-derived secondary structure of proteins (Schneider et al., 1997)) of sequence-structure alignments.

### Definition for different parameters and features

Briefly we define here the different characteristics that ProDFace investigates and we refer the reader to the original paper (Dey et al., 2012) where these properties have been described and benchmarked.

### Interface atoms and residues

For each of the protein–DNA complex, residues residing at the interface are identified. Atoms/residues from both partners that lose >0.1 A^2^ of surface area upon complexation constitute the protein interface. Those residues that have at least one atom fully buried at the interface are referred as core of the interface; the remainder are referred as the rim, has a composition similar to the surface (Chakrabarti and Joël 2002; Janin et al., 2008).

### Identification of conserved residues at the interface

The average sequence entropy for each interface with ‘*n*’ number of residues is calculated as:

〈s〉int=∑s(i)/n

Interface residues with sequence entropy lower than the average (*<s> int* ) were considered as conserved and their total number in each interface is denoted by *N*_*cons*_. *s(i)* is the Shannon entropy of the aligned sequences at position *i* calculated from the MSA of the homologous proteins (Dey et al., 2012).

### Potential hydrogen bond donors

Side-chain groups of positively charged amino acids such as arginine (PDB atom labels: NE, NH1, NH2), histidine (ND1, NE2) and lysine (NZ), as well as of asparagine (ND2), glutamine (NE2), tryptophan (NE1), serine (OG), threonine (OG1) and tyrosine (OH) with accessibility ≥10 Å^2^ are assumed to be capable of getting involved in hydrogen bonding with DNA and their number (*D*_*p*_ ) in each interface/patch is calculated.

### Residue propensity score

Amino acid composition was used to calculate residue propensity score (Bahadur et al., 2004) given by

$$R_{p}=\sum_{i}n_{i}*p_{i}$$

where *n*_*i*_ is the number of residues of type *i* and *p*_*i*_ is its propensity to be in the interface.

### Interface water

Having identified the interface water molecules (those at a distance of ≼4.5Å from both protein and DNA molecule) we find out if they are hydrogen bonded to either of the components, or both (bridging water molecules), or only to other interface water.

The interface residues are spatially clustered using 20 Å as the default value (Biswas et al., 2009) of the distance, which can also be changed. The sequence entropy of the protein chain(s) is calculated either by using the local copy of the HSSP database (Schneider et al., 1997), or by using the MSA supplied by the user. The program uses 30% as the cutoff value for defining close homologs; one can provide even a higher value. In case of a protein-DNA complex whose HSSP file is unavailable (eg. for modelled structure), or the user desires to use his/her own MSA, it is possible to upload the same in FASTA format, the details are provided in the HELP link in the web-tool.

### Test on docking decoys

The physico-chemical features (*R*_*p*_ *, D*_*p*_ and *N*_*cons*_) described above were used to identify the DNA-binding region on the protein component and they performed very well (Dey et al., 2012). In order to further test the efficacy of the features, they were tested on several docking decoys of protein-DNA complexes. The decoy dataset was taken from Varani’s resource–*course docking decoys* (Robertson and Varani 2007) and were reconstructed by running Ftdock (Gabb et al., 1997). The decoy dataset, obtained from Gabriele Varani’s resource, contained 45 different entries. They are provided as FTDock output files, together with the FTDock parameters used in the original docking runs to minimize disk space. The PDB structures from these files were reconstructed using the ‘*build*’ program of the FTDock package. Among them 15 complexes were common to our dataset (Dey et al., 2012), for each of which we generated 100 decoys.

## Results and discussion

### Parameters and output files obtained from ProDFace

All the parameters, which are calculated, have already been defined in (Biswas et al., 2009; Dey et al., 2012) and explanations are also provided in the HELP file. The results are given in the form of tables, plots and downloadable text files. The first table provides information on 18 physicochemical and geometric properties of the whole interface, separately for the protein and the DNA components. The structure of the human NF-kappaB p52 homodimer-DNA complex (Cramer et al., 1997) is used as an example and the values are provided in Table 1. Other tabulated information are: 1) composition of the secondary structural elements, based on which the interface is classified as α, β, αβ or NR (non-regular) (Guharoy and Chakrabarti 2007); 2) dissection of the interface into core and rim regions, followed by the enumeration of the number of atoms, residues (or nucleotides) and interface areas contributed by them; 3) sequence entropies of the core and the rim (Table 2), with the core usually having a lower value (indicating a greater conservation among homologous proteins) than the rim (Guharoy and Chakrabarti 2005).

**TABLE 1:** Parameters for the interface in the human NF-kappaB p52 homodimer-DNA complex (PDB code, 1a3q).

Parameters observed	Protein component	DNA component	Total
Interface area (Å^2^)	1538	1481	3020
Interface area/Surface area	0.05	0.35	0.08
Number of atoms	161	163	324
Number of residues (or nucleotides)	50	22	72
Fraction of non-polar atoms	0.6	0.44	0.52
Non-polar interface area (Å^2^)	766	469	1235
Fraction of fully buried atoms	0.19	0.23	0.21
Residue propensity score	−0.03	—	—
Local density	33	52	—
Number of Potential hydrogen-bond Donors (D_p_)	28	—	—
Number of direct hydrogen-bonds (HBs)	—	—	22
Number of DNA-HOH-Protein H-bond interactions (bridging HB)	—	—	14
Number of HBs involving water and only protein (or DNA)	27	15	42
Number of interface waters	—	—	57
Number of bridging waters	—	—	11
Number of waters H-bonded with only protein or DNA	21	14	35
Number of interface waters H-bonded to other interface waters only	—	—	8
Number of polypeptide segments	7,8	—	15

**TABLE 2:** Sequence entropy data for the interface residues in a subunit of human NF-kappaB p52 (PDB code, 1a3q).

Number of homologs^a^	Number of conserved residues	Mean entropy of core	Mean entropy of rim	Mean sequence entropy of protein
512	13	0.23	0.59	0.78

a Homologous sequences with 30% or more sequence identity were used in multiple sequence alignment obtained from the HSSP database.

Besides providing the physicochemical features of a protein-DNA interface in a given crystal structure (Tables 1, 2), the calculated parameters can also be used in identifying the most likely binding mode from among solutions provided by any docking program or among simulated conformations to represent a protein-DNA complex. Some of the parameters, such as the residue propensity score, potential hydrogen bond donors and the number of evolutionary conserved residues, have been shown to have high discriminatory power (Dey et al., 2012)—the correct solution is expected to have the highest value for all these parameters. While parameters such as the interface area, atoms, the number of hydrogen bonds, etc. would vary depending on the quality/resolution of the structure, others (notably the three mentioned above) may be more useful in locating the gross binding region even for structures of lower resolution, as shown below on applying to a number of docking decoys.

**FIGURE 2:**
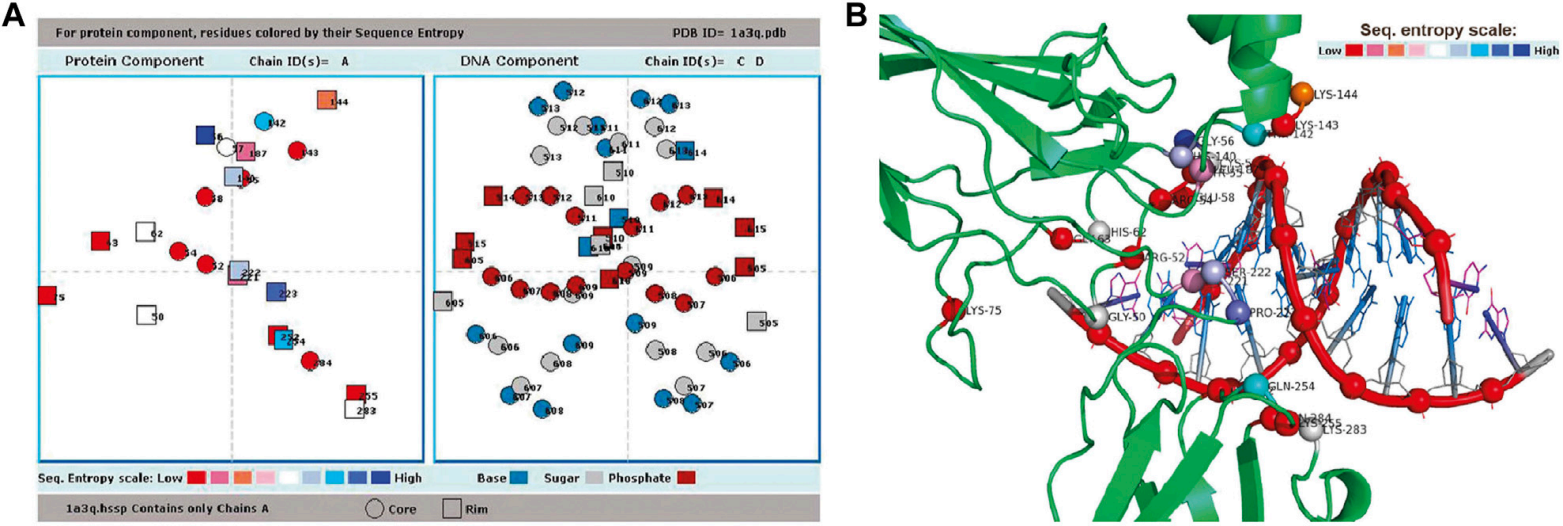
Result page of ProDFace. **(A)** Plot showing relative positions of the residues from one subunit (whose sequence entropies are shown color-coded, and also as circles and squares, depending on their location in core and rim, respectively) and nucleotides (separated into base, sugar and phosphate, shown in three distinct colors, and also distinguished into core and rim) in the file 1a3q.pdb (human NF-kappaB p52 homodimer-DNA complex as input). **(B)** Cartoon representation of human NF-kappaB p52 homodimer-DNA complex (PDB code, 1a3q, only one subunit shown), approximately in the orientation used in Figure 2A for displaying interface residues and nucleotides. Interface C^α^ atoms are shown as spheres and colored according to sequence entropy. DNA backbone is shown in red (phosphate in sphere), whereas base and sugar are shown in blue and gray sticks, respectively. Figure made using pymol (http://www.pymol.org).

Among the different output files generated, the *.int* file contains the list of interacting residues and nucleotides across the interface; *.ent* file contains the Shannon entropies of interface residues representing evolutionary conservation; .*ncons* file contains the list of interface residues having entropies lower than the mean entropy of the whole interface (and these can be designated as conserved residues); .*hbd* file contains information about all possible hydrogen bond donor groups at the interface along with their accessible surfaces areas; *.water* file contains the information about all water-mediated hydrogen bonds between protein and DNA; and .*cont* files contain information about the nucleotides which are in contact with each of the protein residue, and vice versa (Pal et al., 2009). There are plots showing secondary structural segments along the sequence, for each of the protein chains; the interface residues (categorized as core or rim, or belonging to distinct interface patches) are indicated along the sequence. The distribution of the degree of conservation among residues, being colored according to their entropies, is shown in another plot (Figure 2A) by projecting the interface residues down the shortest axis. The same axis is also used to project and display the bound DNA–this enables the visualization of the relative positions of the two components (the cartoon representation of which is given in Figure 2B across the interface.

### Application to docking decoys

All the features mentioned above were calculated for all the docking decoys and these, along with the values for the actual interface, were ranked. The interface was ranked #1 if it occurred within the top 10% of all the decoys (Supplementary Figure S1). There were instances with more than 90% overlap (residue wise) between the decoy and the actual interface. In these cases the feature incorporating amino acid composition (*R*_*p*_), which has been by far the best discriminator (Dey et al., 2012), did not perform well, identifying the interface as rank #1 in only 53.3% cases (Supplementary Figure S1C). Interestingly, even in these cases the hydrogen bond donor potential (*D*_*p*_) performed notably well, discriminating the actual interface from a decoy having 94% overlapping residues. The reason behind this may be *D*_*p*_ is not merely a frequency of possible donor groups at the binding region, but an area criterion is also incorporated in its definition (only those with accessibility ≥10 Å^2^ are counted) (Dey et al., 2012). In these decoys even if the interface is the same as the real one in terms of residues, the appropriate donor atoms (which account to *D*_*p*_) may be missing. As a result, for the real binding region there are more number of solvent exposed donor atoms capable of forming hydrogen bonds with DNA.

To see if the performance of *R*_*p*_ improves on removing the overlaps, we gradually removed the overlaps and re-ranked all at various percentages of overlaps, such as 50, 20, 10 and 0%. We found that at 10% overlap the performance of *R*_*p*_ improved considerably and it could identify the actual interface among the decoys, ranking it as 1 in 66% cases (Supplementary Figure S1A). At 0% overlap (i.e. no common residues) *R*_*p*_ performed the best, identifying 73% of the actual interfaces correctly as rank 1, with all but one in the top 3 ranks (Supplementary Figure S2). The lone entry (PDB: 1je8) for which *R*_*p*_ failed was found to have a very negative propensity score for the real interface itself and hence it could not be distinguished by amino acid propensity criterion, whereas it was ranked 1 by *D*_*p*_. *D*_*p*_ identified 86% interfaces as rank 1 in the category of 0% overlap as well. There is only one entry (PDB: 2bop) for which *D*_*p*_ could not rank the actual interface in top 3, this however was ranked 1 by *R*_*p*_. So, it seems that *D*_*p*_ and *R*_*p*_ complement each other and in all the entries the interface patch is ranked 1 by more than one parameter; as such there is no single entry whose interface cannot be discriminated from the decoys by any of the features defined by us. Performance of *N*_*cons*_ is also comparable to that of *R*_*p*_ but when overall top 3 rankings are considered *R*_*p*_ outperforms *N*_*cons*_ (Supplementary Figure S2). All the rankings at different percentage cutoffs of overlap—10, 50 and 100% are provided in Supplementary Figure S1. Results for 20 and 10% are similar, hence only data for 10% is shown.

## Conclusion

We describe a web-tool, ProDFace that enables researchers to upload structures obtained from experimental methods, docking programs, or derived from simulation, for analysis. The program derives the overall characteristics of the binding region of a protein-DNA complex structure, in particular of the protein component.

Targeting protein-DNA complexes with small molecule inhibitors is difficult compared to protein-protein complexes. Recent developments in computer-aided drug discovery approaches are using key oncogenic transcription factors and have developed candidate inhibitors targeting the DNA binding region, are presently under clinical trials (Radaeva et al., 2021). Now, with the proteome wide increase in macromolecule structural data initiated by ALPHAFOLD (Jumper et al., 2021), it is anticipated that they would also come up with protein-DNA complex models. In all these cases, ProDFace pipeline can be efficiently used to study binding region characteristics specifically interactions, geometry, hydration and sequence conservation.

Further, understanding the various features that characterize protein-DNA interfaces would help us develop empirical algorithms that can identify the DNA-binding patch in protein structures (Jones et al., 2003; Stawiski et al., 2003; Paillard and Lavery 2004; Dey et al., 2012). The top solutions of protein-DNA complex structures obtained from the available docking and prediction programs can be cross validated with the help of ProDFace. Docking decoys are normally used to test complex scoring functions that are implemented in the docking algorithms. Our simple single statistical features performed quite satisfactorily in comparison to the various scoring functions that are rigorously generated. Thus the properties generated by the web-tool can be crucial in developing scoring functions for protein-DNA docking algorithms that are still in the developing stage. Likewise, the ProDFace tool can also be used to judge the stability of any protein-DNA complex conformation obtained from all-atom simulations. Present version of ProDFace is built dedicatedly for the analysis of protein-dsDNA complexes. Our approach, however, is general enough and currently the web-tool also supports structures of proteins bound to RNA or ssDNA for the analysis. In the future, we will include specific features for protein-RNA as well as protein-ssDNA complexes. Furthermore, the program presently takes a single input, batch upload service of multiple structures will also be one of the future implementations.

## Data Availability

Publicly available datasets were analyzed in this study. This data can be found here: We refer the reader to read the original paper (Dey et al., 2012; PMID: 22641851) where the properties used here, have been described and benchmarked. Also, important source codes of the program are available at https://github.com/sdeyLab-IITJ/ProDFace
