# Supplementary material for: ProDFace: A web-tool for the dissection of protein-DNA interfaces
Source: Front Mol Biosci. 2022 Sep 6;9:978310. doi: 10.3389/fmolb.2022.978310 (PMC9486321; doi:10.3389/fmolb.2022.978310)
Supplement: Supplementary file 1 [file DataSheet1.PDF]

Supplementary to
ProDFace: A Web-tool for the Dissection of Protein-DNA Interfaces

Arumay Pal[1], Pinak Chakrabarti[2], Sucharita Dey[3]*

[1]School of Bioengineering, Vellore Institute of Technology, Bhopal, India

[2]Department of Biochemistry, Bose Institute, P1/12 CIT Scheme VIIM, Kolkata 700054, India

[3]Indian Institute of Technology Jodhpur, NH 62 Nagaur Road, Karwar, 342037, India

Figure S1.

(a)

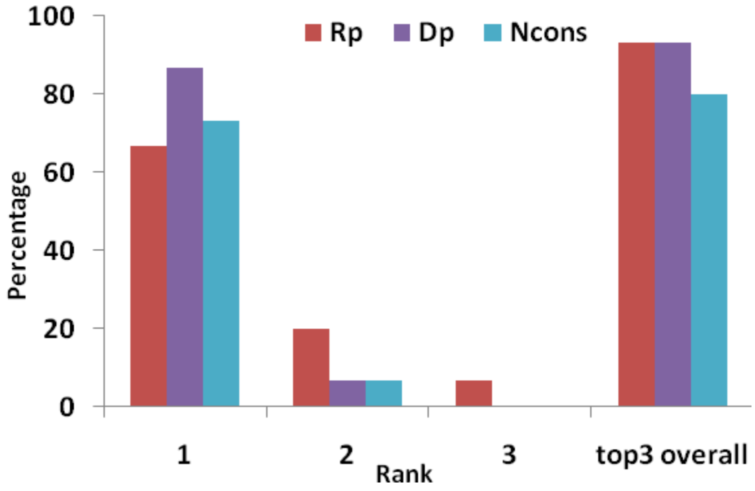

(b)

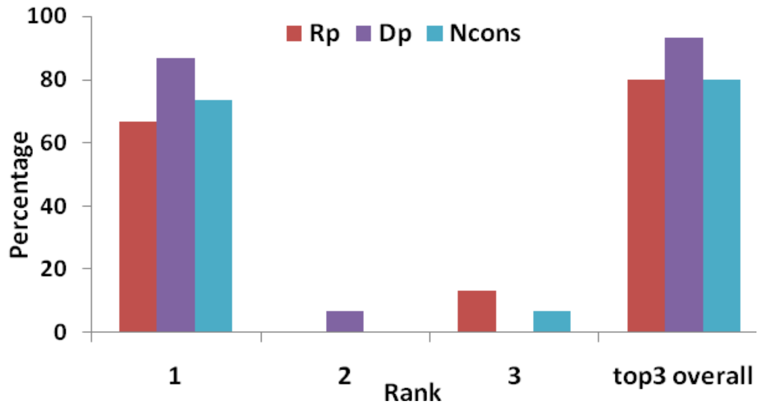

(c)

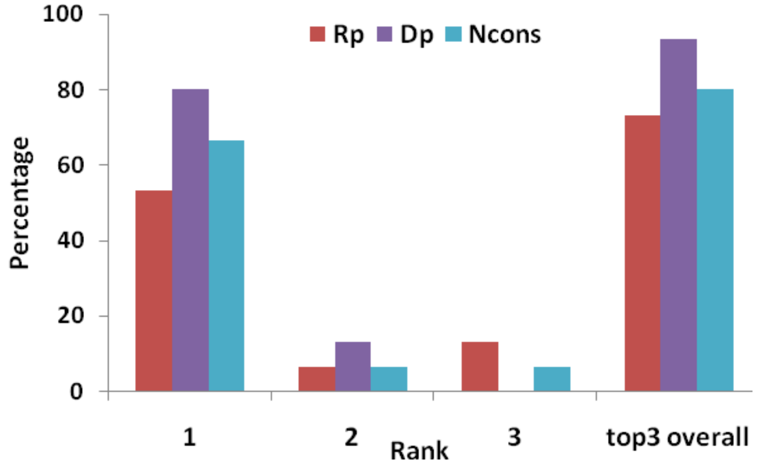

Figure S1. Distribution of the rank of the interface in DNA-binding proteins relative to the docking decoys. Only top 3 ranks are used, and the 'overall' indicates that the interface is within the top 3 ranks. (a)-(c) are for decoys having up to 10, 50 and 100% overlap with the actual binding site, respectively.

Figure S2.

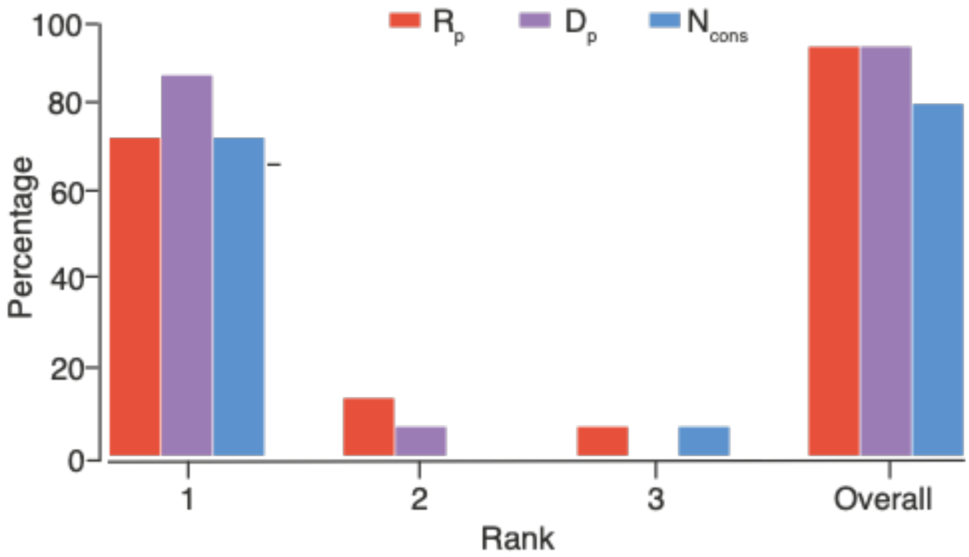

Figure S2. Distribution of the rank of the interface in DNA-binding proteins relative to the docking decoys (having 0% overlap with the actual binding site). Only top 3 ranks are used, and the 'overall' indicates that the interface is within the top 3 ranks.
